# Common ground, complementary approaches: adapting the Housing First model for domestic violence survivors

**DOI:** 10.1080/08882746.2017.1323305

**Published:** 2017-06-12

**Authors:** Cris M. Sullivan, Linda Olsen

**Affiliations:** a Psychology Department, Michigan State University, East Lansing, MI, USA; b Washington State Coalition Against Domestic Violence, Seattle, WA, USA

**Keywords:** Intimate partner violence, domestic violence, homeless, housing first

## Abstract

The Housing First model has been shown to be a highly effective approach to achieving permanent housing for chronically homeless individuals with serious mental illness and chemical dependency. There are numerous components of the model that lend themselves toward achieving similar goals for homeless domestic violence (DV) survivors and their children. A leading cause of homelessness for women, many of whom are mothers, is DV. This article describes the commonalities between the Housing First model and the tenets of DV victim advocacy work and explores how Housing First can be adapted to effectively achieve safe and stable housing for DV survivors and their children. Preliminary evidence for the adapted model – termed Domestic Violence Housing First – is provided, and policy implications are discussed.

Homelessness continues to be a serious and devastating social issue plaguing the United States. It has been estimated that approximately 3 million adults and over a million school-aged children are homeless in any given year (Kilgore, 2013; National Center for Homeless Education, 2015; Perlman, Willard, Herbers, Cutuli, & Eyrich Garg, 2014). Numerous factors increase one’s risk for becoming homeless, including the lack of affordable housing, living in poverty, experiencing serious mental illness (SMI), and for women, domestic violence (DV) victimization (Joint Center for Housing Studies, Harvard University, 2013; National Alliance to End Homelessness, 2015; Paveo et al., 2007).

The Housing First model was originally created as an alternative to the more traditional “housing ready” model, which expected homeless individuals to deal with their mental health issues and chemical dependencies before being offered stable housing (Padgett, Henwood, & Tsemberis, 2016; Pleace & Bretherton, 2013). The traditional model for helping people with SMI and/or chemical dependency (CD) was communal residential treatment, followed by supported living, and then ultimately independent living (Carling, 1990; Pleace & Bretherton, 2013; Ridgway & Zipple, 1990). This has been referred to as linear residential treatment or the staircase model and has been widely criticized for being overly controlling, resulting in many people dropping out (Padgett et al., 2016).

The Housing First model was based on the presumption that helping people obtain stable housing before addressing other concerns makes dealing with these other issues easier, and the evidence has strongly supported this claim (Padgett et al., 2016; Tsemberis, 2010). However, the model has relied on the availability of a stream of government funding targeted toward chronically homeless people with SMI and chemical dependencies (McKinney–Vento Continuum of Care funding), and this funding is not readily available to other populations. Further, most of the evidence for Housing First’s success has come from studies with single, primarily male, adults. Therefore, a number of adaptations may be necessary in order to effectively use this model with those who are homeless for reasons beyond SMI or chemical dependencies.

For women, being the victim of DV is a leading cause of homelessness (Jasinski, Wesely, Mustaine, & Wright, 2002; Kannah, Singh, Nemil, & Best, 1992; Wilder Research Center, 2007). Housing instability is four times more likely for women who have experienced DV compared to other women (Pavao et al., 2007), and approximately 25% of homeless women have noted DV as being a major contributor to their homelessness (Jasinski et al., 2002; Wilder Research Center, 2007). DV victimization has both direct and indirect pathways to homelessness. Many abusers intentionally destroy their victims’ economic and housing stability by ruining their credit, stealing their money, destroying their property, or preventing them from working, as a means of trapping them in the relationship (Adams, Sullivan, Bybee, & Greeson, 2008; Adams, Tolman, Bybee, Sullivan, & Kennedy, 2012; Hahn & Postmus, 2014). Further, the DV itself often leads to injuries, depression, posttraumatic stress disorder (PTSD), and frequent absences from jobs or school, all of which can then contribute to homelessness (Adams, Bybee, Tolman, Sullivan, & Kennedy, 2013; Lacey, McPherson, Samuel, Sears, & Head, 2013). Sometimes, DV survivors flee their homes and find new immediate housing, only to realize they can not afford it on their own (Galano, Hunter, Howell, Miller, & Graham-Bermann, 2013). All of these factors can make achieving safe and stable housing difficult, especially since affordable housing in the United States is scarce and continues to decline (Joint Center for Housing Studies, Harvard University, 2013; National Alliance to End Homelessness, 2015).

Advocates for DV survivors are often faced with helping their clients locate safe and affordable housing, a daunting challenge in most parts of the country where affordable housing is scarce or nonexistent (Joint Center for Housing Studies, Harvard University, 2013; National Alliance to End Homelessness, 2015). These advocates base their work on many of the same principles guiding the Housing First model. Shared principles include (1) viewing housing as a basic right; (2) treating clients with respect, warmth, and compassion; (3) working with people as long as they need; (4) moving people into independent housing; and (5) separating housing from services (see Table 1). There are an additional three Housing First tenets that suggest areas where the model could be adapted to be relevant for DV survivors, and these are discussed next.

**Table 1.** Shared and similar principles between the Pathways Housing First model and domestic violence victim advocacy.^2.^

Housing First model tenets (Tsemberis, 2010, p. 18)	Domestic violence victim advocacy tenets^2^
*Shared principles*	
Housing is a basic human right	Housing is a basic human right
Respect, warmth, and compassion for all clients	Respect, warmth, and compassion for all clients
Commitment to working with clients for as long as they need	Commitment to working with clients for as long as they need
Scattered site housing; independent apartments	Scattered site housing; independent apartments (and communal shelters)
Separation of housing and services	Separation of housing and services
*Similar principles*	
Consumer choice and self-determination	Strength-based, empowerment focus
Harm reduction	Safety planning
Recovery orientation	Orientation toward social and emotional well-being
*Explicit principles of domestic violence victim advocacy*	
	Community engagement, systems change
	Trauma-informed practice

Where the Housing First model refers to “consumer choice and self-determination” (Tsemberis, 2010, p. 18), DV advocates would refer to “empowering practice” (Cattaneo & Chapman, 2010; Kasturirangan, 2008; Kulkarni, Bell, & McDaniel Rhodes, 2012). This involves behaving with survivors in ways that not only honor their agency but that will also increase their power in personal, interpersonal, and political arenas (Gutierrez & Lewis, 1999; Gutierrez, Parson, & Cox, 1998; Sullivan, 2006). Empowerment practice in general is a helping relationship through which the advocate (or other form of help provider such as a case manager) shares power with the participant and is a facilitator, not a director, of services. In the DV context, the advocate works *with* the survivor to facilitate their access to knowledge, skills, supports, and resources that will enhance their personal power. Advocates’ analysis of DV is that abusers use violence and control to rob victims of their power to determine their own destiny. Therefore, advocates intentionally work to restore that sense of power by offering information and choices, while recognizing and supporting survivors’ strengths and decisions.

While empowering practice *includes* “consumer choice and self-determination,” DV advocates recognize that advancing survivors’ empowerment entails more than influencing the way they think and feel about themselves and their abilities (Goodman & Epstein, 2008; Kulkarni et al., 2012; Song, 2012; Sullivan, 2006). While self-determination is an important construct, and central to the concept of empowerment, it is not synonymous with actually *increasing* survivors’ power in interpersonal, social, and political spheres (Cattaneo & Chapman, 2010). Staples (1990) perhaps summarized it best: “In addition to transformations in consciousness, beliefs, and attitudes, empowerment requires *practical knowledge, solid information, real competencies, concrete skills, material resources, genuine opportunities, and tangible results*” (p. 38; emphasis added).

The second Housing First component that requires adapting for the DV context is harm reduction. Harm reduction involves helping clients minimize risky behaviors rather than mandating full compliance with either chemical abstinence or, in the case of people with SMI, taking prescribed medication. DV advocates only follow this principle for those survivors who also have SMI or who are chemically dependent; a core activity they do engage in with all survivors, however, is safety planning (Davies & Lyon, 2014; Goodkind, Sullivan, & Bybee, 2004). For many DV survivors, the abuse or fear of future abuse is ongoing, regardless of their relationship status (Fleury, Sullivan, & Bybee, 2000). Advocates draw on their understanding of the dynamics of DV to consider how the abuse is impacting other issues survivors are dealing with, including their housing, economic independence, parenting, custody, legal issues, immigration, and social support. In other words, when a survivor is contending with ongoing DV, safety issues need to be continually addressed along with other concerns.

While it is understood that safety planning efforts may or may not be successful, given the individual circumstances surrounding each incident of abuse and the reality that the perpetrator is ultimately responsible for whether abuse occurs, a variety of strategies are discussed with survivors to help them decide what might or might not reduce their future risk of abuse. Safety planning is therefore related to harm reduction, but with the understanding that the advocate is working with the survivor to reduce someone else’s harmful acts, and not the survivor’s “risky” behaviors.

The third Housing First tenet that requires some modification is the model’s “recovery orientation” – working with consumers in ways that will lead to their full participation in their communities (Tsemberis, 2010). This tenet stems from the model’s focus on persons with SMI, who have often been segregated from their communities and viewed as incapable of full integration. For DV victim advocates, the focus is on increasing survivors’ access to resources, opportunities, and supports needed for them to achieve social and emotional well-being (Sullivan, 2016).

In addition to the eight tenets just discussed (five shared, three similar), DV advocates are guided by two additional principles not explicitly stated in the Housing First model^1^: (1) engaging in trauma-informed practice (Goodman, Fauci, Sullivan, DiGiovanni, & Wilson, 2016; Goodman et al., 2016) and (2) systems change and community engagement (Goodman & Epstein, 2008; Sullivan, 2016). Trauma-informed practice is grounded in an understanding that DV is an ongoing pattern of coercive control maintained through physical, psychological, sexual, and/or economic abuse that varies in severity and chronicity. It is not surprising, then, that DV survivors’ responses to this victimization vary as well. Many victims of DV recover relatively quickly from the experience, particularly if the abuse is shorter in duration and less severe, and they have access to resources and support (Bonanno, 2004). Others, particularly those who experience more frequent or severe abuse, may develop symptoms that make daily functioning more difficult, including depression, substance abuse, anxiety, and PTSD (Bennice, Resick, Mechanic, & Astin, 2003; Anderson, 2009; Dillon, Hussain, Loxton, & Rahman, 2013). Survivors who are having trouble concentrating, who are in a state of constant high anxiety, or who are not sleeping (just to name a few examples) may find it temporarily difficult to make decisions or feel emotionally in control of their lives. Advocates strive to provide survivors and their children with the time, space, and supports needed to heal from traumas that may be impeding their ability to fully reintegrate into their communities and to achieve social and emotional well-being (Warshaw, Sullivan, & Rivera, 2013).

Another core principle of DV advocacy involves systems change and community engagement. Recognizing that one’s social and emotional well-being is not independent from community-level factors, advocates do not focus solely on working with individual survivors to improve their individual situations. They engage in a variety of efforts to create communities that hold offenders accountable, promote justice, and that provide adequate resources and opportunities for the survivors they work with and all community members (Sullivan, 2016). This involves building and sustaining strong community partnerships as well as working to change systems that are ineffective, inaccessible, or oppressive.

## The creation of the domestic violence Housing First model

In light of the many shared principles between Housing First and DV advocacy work, and given the promise of the original Housing First model, in 2009 the Bill & Melinda Gates Foundation approached the Washington State Coalition Against Domestic Violence (WSCADV) with a funding proposal to adapt the model for DV survivors. Termed Domestic Violence Housing First (or DV Housing First, or DVHF), the program was designed to increase survivors’ access to and retention of not just stable but *safe* housing (Mbilinyi, 2015). The model was evaluated through a 5-year pilot involving 13 DV agencies from diverse areas within Washington State. Seven of the nine agencies were in rural areas, where resources were fewer and the need for creative solutions much greater. Three of the rural programs were tribal and located on reservations. Another rural program served many migrant Latina farmworkers. One of the two urban programs was a culturally specific program, serving immigrants and refugees from Southeast Asia, South Asia, Africa, and Eastern Europe. Out of the 681 survivor households participating in the evaluation, 35% were Native American/Alaska Native, 22% were immigrant/refugee, and 12% were Hispanic or Latino/a. Forty-six languages were spoken in the survivors’ homes. Half had a household monthly income of $800 or less. The majority of survivors (75%) had children, with a combined total of 937 children living in their households (Mbilinyi, 2015).

**Figure 1.**
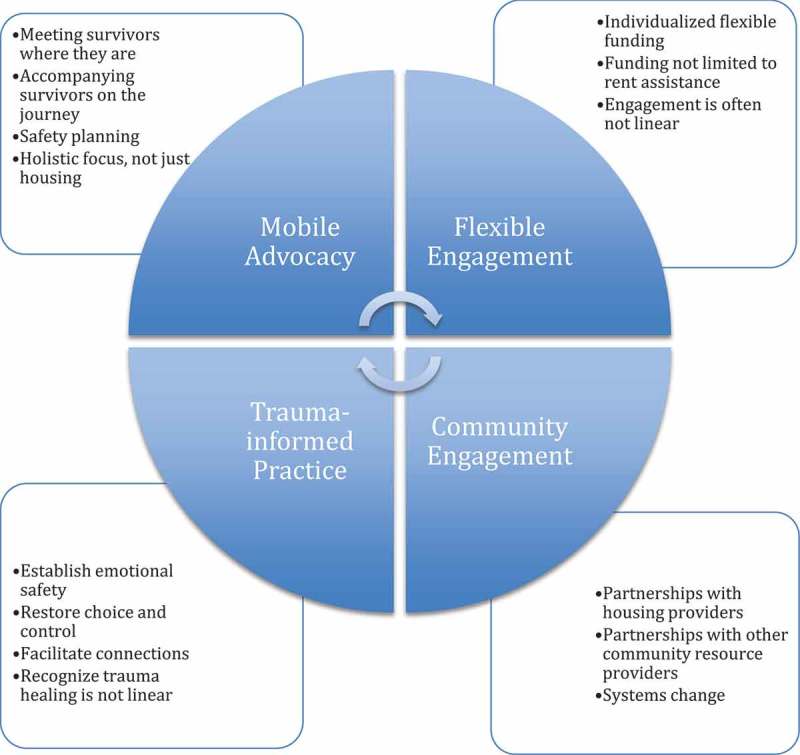
Tenets of domestic violence Housing First.

Program records indicated that 96% of the families receiving DV Housing First retained their housing at 18 months. Focus group data with survivors revealed additional positive outcomes for families, including increased safety, improved health and well-being, and restored dignity. Lessons learned from the pilot led WSCADV to identify four key service pillars of the model: (1) survivor-driven, mobile advocacy; (2) flexible engagement, including flexible funding; (3) trauma-informed practice; and (4) community engagement (see Figure 1). The philosophy guiding the model is that survivors and their children need individualized and flexible levels of assistance. The four pillars are described next.

### Survivor-driven, mobile advocacy

A critical component of the model is that advocates focus on addressing the needs identified by survivors rather than on predetermined needs promoted by agencies. Advocates are also mobile, meeting survivors where it is safe and convenient for them, and advocacy continues as long as survivors need support. Advocates are aware of the myriad ways that abusers sabotage survivors’ economic and housing stability – even after the relationship has ended – and they mobilize multiple resources and community supports to prevent or counter these abusive activities. In addition to advocating for survivors in other aspects of their lives (e.g., employment, immigration, health, children’s needs) and engaging in ongoing safety planning, advocates work proactively and creatively with survivors to obtain housing stability. This may involve helping a survivor safely retain her current housing or helping find new affordable housing. Advocates are proactive and creative, accompanying survivors to housing appointments, acting as liaisons with landlords, and negotiating leases.

### Flexible engagement, including flexible funding

Many survivors need not only proactive advocacy to obtain safe and stable housing but also temporary financial assistance to get back on their feet. They may need assistance with issues viewed as directly related to housing: a security deposit and temporary rental assistance, help clearing up rent arrears (often intentionally incurred by the abuser), or help with utility bills. Often, though, survivors need funds that may not be viewed by others as impacting housing but that advocates recognize are critical to housing stability: for example, help repairing their cars so they do not lose their jobs, help expunging a prior conviction that is preventing them from obtaining government-funded housing, or help repairing bad credit (often destroyed by the abuser). Funds are targeted to support survivors so they can rebuild their lives, including covering childcare costs, transportation, school supplies, uniforms and permits required for employment, as well as time-limited and flexible rental assistance (Mbilinyi, 2015; Sullivan, Bomsta, & Hacskaylo, 2016).

The idea of providing flexible funding with individualized advocacy is similar to but distinct from the idea of “progressive engagement” promoted by the US Interagency Council on Homelessness and the US Department of Housing and Urban Development. Progressive engagement involves providing every client with a very small and brief amount of assistance (“light touch”) and then providing additional funds and other supports only as needed to select clients over time (Culhane, Metraux, & Byrne, 2011; Levitt, 2015). While this is sometimes appropriate, the DVHF model promotes the idea of “flexible engagement,” tailoring financial and support assistance to the individual needs of each survivor. For some survivors, that may be a small amount of funding followed by a check-in to see if additional funds are needed (the traditional progressive engagement model). For others, however, a larger immediate investment may be necessary and sufficient to stabilize someone’s housing, and for others, assistance will fluctuate with crises (sometimes caused by the abuser when a survivor initially becomes stable). This flexibility is a critical component of the DV Housing First model and is consistent with the philosophy of DV advocacy to provide individualized, survivor-centered services (Davies & Lyon, 2014; Goodman & Epstein, 2008).

### Trauma-informed practice

Given the traumatic nature of DV, as well as the likelihood that DV survivors have also experienced other lifetime traumas such as child abuse and sexual abuse (Campbell, Greeson, Bybee, & Raja, 2008), a critical tenet of DV Housing First is to engage in trauma-informed practice. These practices include (1) establishing emotional safety, (2) restoring choice and control, (3) facilitating survivors’ connections to community supports, (4) supporting coping, (5) responding to identity and context, and (6) building strengths (Anderson, 2009; Goodman, Sullivan, et al., 2016; Harris & Fallot, 2001). Understanding and appropriately responding to trauma reactions is especially important when helping survivors obtain and sustain housing, as sometimes these responses manifest after initial stability is attained (Ferencik & Ramirez-Hammond, 2013; Horesh, Solomon, Zerach, & Ein-Dor, 2011). Sometimes, trauma reactions such as depression, immobility, or PTSD are suppressed while a survivor is intently focused on the task of securing housing for themselves and their children. Once that housing is obtained, however, and an initial calm is established, the survivor is “safe” to experience the overwhelming feelings related to their trauma. Without a knowledgeable and supportive advocate available to them to help them through this crisis, the housing that the survivor has worked so hard to secure can be jeopardized.

### Community engagement

Advocates also proactively engage those people in the community who can help support the safety, stability, and well-being of survivors. This includes engaging with health-care professionals, law enforcement and the legal systems, educators and school administrators, religious and spiritual leaders, and others. With specific regard to obtaining housing, advocates forge mutually beneficial relationships with landlords, city officials, and housing councils to obtain vouchers or rental agreements on behalf of DV survivors. Through these relationships, advocates not only obtain housing for individual survivors, but they change and improve the way communities respond to DV overall.

## The importance of having multiple housing options for DV survivors

As noted earlier, the Pathways Housing First model relies heavily on obtaining permanent housing vouchers for clients with SMI. Such vouchers have been found to be the most desired as well as the most successful option for homeless families in general (Gubits et al., 2015), but they are also a very expensive option and there are not enough vouchers to meet the need. They are also not necessarily what is needed by many DV survivors. DV Housing First, then, focuses on matching each DV survivor with the housing option best suited to their need. For some survivors, this means receiving immediate financial and support services to stay in their current homes, thus avoiding homelessness altogether (Sullivan, Bomsta, and Hacskaylo, 2016). Interestingly, half of the survivors participating in the DV Housing First pilot expressed the desire to remain safely in either their current home or in a home they obtained when immediately fleeing an abuser but which they could not manage or afford long term (Mbilinyi, 2015). While eviction-prevention efforts or advocacy with landlords were often helpful with this option, providing flexible financial assistance was also the key. Such assistance might include paying back rent that the abuser failed to pay, fixing property damaged by the abuser, repairing the survivor’s car so they do not lose their job, paying for a security system, or any number of issues that, but for the availability of a flexible pot of funds, could lead to homelessness.

In short, DV advocates need to work with survivors to identify the most appropriate short- and long-term housing options that they desire and that are available to them. DV survivors who have had relatively stable economic resources in the past and who have minimal obstacles to sustaining stable housing may need relatively brief assistance obtaining safe and secure housing. In these cases, safety planning and information may be all a survivor requires. At the other end of the continuum, there are DV survivors with multiple, complex issues that will require longer term and more sustained supports from advocates. These may include survivors who are non-English-speaking refugees or immigrants who had relied on the abuser for their economic and social support, or those with criminal records (often related to the abuser’s coercion; Richie, 2012) and/or SMI. Low-income women of color are at greater risk of experiencing victimization as well as experiencing the mental and physical health sequelae resulting from it (Kennedy et al., 2012), and DV victimization has been linked to increased CD (Bonomi et al., 2006; Humphreys, Regan, River, & Thiara, 2005). Survivors with extremely complex issues are sometimes unable to sustain housing past the 24 months designated by Rapid Re-Housing or Transitional Housing programs, and in these cases, long-term subsidies with voluntary services need to be available.

## Promising evidence for the DV Housing First model

The development of the DV Housing First model was informed by both practice-based evidence and evidence-based practice. A large, randomized controlled trial conducted in the 1990s had established that mobile advocacy leads to improvements in DV survivors’ ability to access community resources (including housing), social support, safety from abuse, and overall quality of life (Bybee & Sullivan, 2002; Sullivan & Bybee, 1999). Building on this earlier work, Niolon and colleagues (2009) longitudinally examined the role of housing stability in preventing revictimization and reducing negative outcomes for DV survivors and their children. That study, which included an examination of mobile advocacy and housing supports over time, found quite positive changes in women’s and children’s lives over 18 months. Women who were homeless or at high risk for homelessness when entering the study reported greater housing stability, higher quality of life, fewer absences from work, greater job stability, higher income, fewer problems with alcohol/drugs, less depression, and less PTSD over time. Their children missed fewer days of school, had better academic performance, and fewer behavioral problems over time.

WSCADV’s evaluation of the DV Housing First model was similarly promising. The majority of families in both rural and urban communities reported being effective at accessing and retaining housing at 6, 12, and 18 months after program entry. Participants also reported increased safety and well-being. More rigorous evidence is needed to examine the impact of this model (and is currently in process), but evidence to date is quite promising.

## Looking forward

Given the many similarities between the generic Housing First approach and the tenets underlying advocacy with DV survivors, adapting the Housing First model to meet the needs of this population could go a long way toward ending family homelessness (Jasinski et al., 2002; Pavao et al., 2007). The two approaches already share the important principles that housing is a basic human right, clients must be treated with respect and dignity, we must work with people as long as they need assistance, and services must be disentangled from the right to independent housing. Adaptations take into account and address issues specific to DV survivors. For example, a number of barriers presented by survivors are rooted in ongoing safety concerns and abuser sabotage. When case managers lack an understanding of these multifaceted issues, they may not provide the most appropriate assistance that will lead to safe and stable housing.

An effective model for stabilizing housing for DV survivors must also attend to the impacts of trauma on both survivors and their children. Trauma responses, which sometimes include CD, increase in severity when the abuse reaches back into a survivor’s childhood and continues through adult relationships (Carlson, McNutt, & Choi, 2003; Seedat, Stein, & Forde, 2005). While most survivors have the resiliency to move quickly on with their lives once a sense of safety has been restored, many others need extensive periods of time to heal and to regain or gain confidence in their ability to return to school, complete job training, and sustain employment at a living wage job adequate to pay for market rate housing.

A final but critical point to make is that no model will be widely successful without the creation of additional affordable housing in this country. In too many communities, affordable housing is simply out of the reach of the typical American (Joint Center for Housing Studies, Harvard University, 2013; National Alliance to End Homelessness, 2015). Until this crisis is abated, even the best approaches will be severely limited in what they can achieve. The reduction of homelessness requires a multipronged solution focused at multiple levels. At the individual and community level, the DVHF approach holds great promise for assisting survivors to obtain safe and stable housing over time for themselves and their families.

## Notes

1. While the Housing First model does not explicitly include attending to trauma or systems change (Tsemberis, 2010, p. 18), both are often implied or alluded to, and many programs operating from the model include one or both in their practice.

2. These common tenets have been drawn from multiple sources, including Davies and Lyon (2014), Goodman and Epstein (2008), and Sullivan (2016).
